# Comparison of Lesion Detection and Quantification in MAP Reconstruction with Gaussian and Non-Gaussian Priors

**DOI:** 10.1155/IJBI/2006/87567

**Published:** 2006-06-29

**Authors:** Jinyi Qi

**Affiliations:** Department of Biomedical Engineering, University of California, Davis, CA 95616, USA

## Abstract

Statistical image reconstruction methods based on maximum a posteriori (MAP) principle have been developed for emission tomography. The prior distribution of the unknown image plays an important role in MAP reconstruction. The most commonly used prior are Gaussian priors, whose logarithm has a quadratic form. Gaussian priors are relatively easy to analyze. It has been shown that the effect of a Gaussian prior can be approximated by linear filtering a maximum likelihood (ML) reconstruction. As a result, sharp edges in reconstructed images are not preserved. To preserve sharp transitions, non-Gaussian priors have been proposed. However, their effect on clinical tasks is less obvious. In this paper, we compare MAP reconstruction with Gaussian and non-Gaussian priors for lesion detection and region of interest quantification using computer simulation. We evaluate three representative priors: Gaussian prior, Huber prior, and Geman-McClure prior. We simulate imaging a prostate tumor using positron emission tomography (PET). The detectability of a known tumor in either a fixed background or a random background is measured using a channelized Hotelling observer. The bias-variance tradeoff curves are calculated for quantification of the total tumor activity. The results show that for the detection and quantification tasks, the Gaussian prior is as effective as non-Gaussian priors.


Hindawi Publishing Corporation
International Journal of Biomedical Imaging
Volume 2006, Article ID 87567, Pages 1–10
DOI 10.1155/IJBI/2006/87567

### Comparison of Lesion Detection and Quantification in MAP Reconstruction with Gaussian and Non-Gaussian Priors

**Jinyi Qi**

*Department of Biomedical Engineering, University of California, Davis, CA 95616, USA*

Received 21 December 2005; Revised 13 April 2006; Accepted 19 April 2006

Statistical image reconstruction methods based on maximum a posteriori (MAP) principle have been developed for emission tomography. The prior distribution of the unknown image plays an important role in MAP reconstruction. The most commonly used prior are Gaussian priors, whose logarithm has a quadratic form. Gaussian priors are relatively easy to analyze. It has been shown that the effect of a Gaussian prior can be approximated by linear filtering a maximum likelihood (ML) reconstruction. As a result, sharp edges in reconstructed images are not preserved. To preserve sharp transitions, non-Gaussian priors have been proposed. However, their effect on clinical tasks is less obvious. In this paper, we compare MAP reconstruction with Gaussian and non-Gaussian priors for lesion detection and region of interest quantification using computer simulation. We evaluate three representative priors: Gaussian prior, Huber prior, and Geman-McClure prior. We simulate imaging a prostate tumor using positron emission tomography (PET). The detectability of a known tumor in either a fixed background or a random background is measured using a channelized Hotelling observer. The bias-variance tradeoff curves are calculated for quantification of the total tumor activity. The results show that for the detection and quantification tasks, the Gaussian prior is as effective as non-Gaussian priors.

Copyright © 2006 Jinyi Qi. This is an open access article distributed under the Creative Commons Attribution License, which permits unrestricted use, distribution, and reproduction in any medium, provided the original work is properly cited.

### 1. INTRODUCTION

Statistical image reconstruction methods have been developed for emission tomography to improve the signal-to-noise ratio (SNR), for example, [1–3]. The most popular maximum-likelihood (ML) reconstruction algorithm is the expectation-maximization (EM) algorithm [1, 4]. However, the ML estimate can be very noisy because emission tomography is an ill-posed problem. Hence some form of regularization is needed to obtain a useful image. Bayesian methods regularize the solution by using a prior probability distribution on the image. The prior probability distribution plays an important role in Bayesian image reconstruction. The most commonly used prior is the Gaussian prior which strongly discourages sharp transitions in images. To preserve edges, non-Gaussian priors have been proposed [3, 5, 6]. Empirical results showed that images reconstructed with edge-preserving non-Gaussian priors have less mean-squared error than those reconstructed with the Gaussian prior. However, the effect of edge-preserving priors on clinical tasks is not obvious.

Gifford et al. [7] compared a quadratic penalty function with Huber penalty functions in a penalized-EM algorithm for tumor detection. However, the comparison was done as a function of iteration, so the result is algorithm-dependent. Nuyts and Michel [8] compared maximum a posteriori (MAP) reconstruction with a relative difference prior to post-smoothed ML reconstruction for hot lesion detection and found similar performance between MAP and post-smoothed ML reconstructions. In this paper we evaluate the performance of Bayesian image reconstructions with the Gaussian and non-Gaussian priors for hot lesion detection and region of interest quantification, which are two major clinical applications of emission tomography. Some preliminary results were reported in [9].

This paper is organized as follows. We briefly review Bayesian image reconstruction and different prior functions in Section 2. The methods for evaluating image quality are described in Section 3. The results are presented in Section 4. Finally, the conclusion is drawn in Section 5.

### 2. BAYESIAN IMAGE RECONSTRUCTION

In emission tomography data are well modeled as a collection of independent Poisson random variables with the mean $\bar{\mathbf{y}} \in \mathbb{R}^{M\times 1}$ related to the unknown image, $\mathbf{x} \in \mathbb{R}^{N\times 1}$, through an

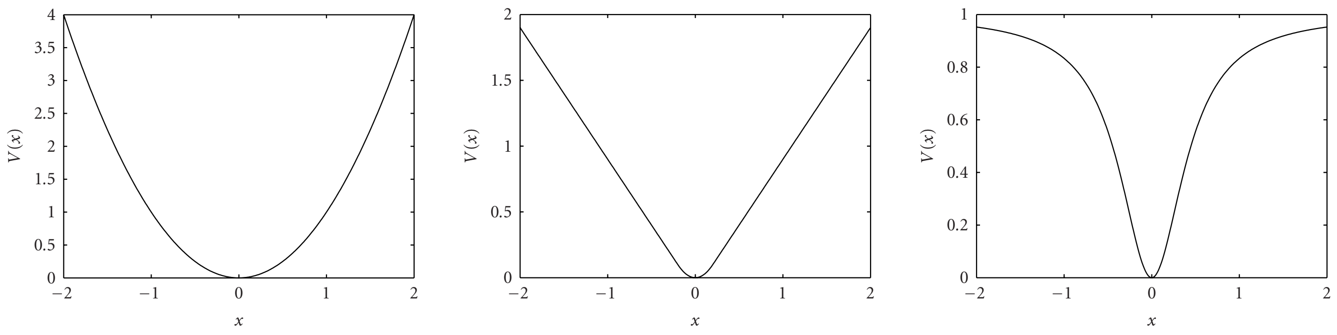

(a) Gaussian (b) Huber (c) Geman-McClure

FIGURE 1: The potential functions of the Gaussian, Huber, and Geman-McClure priors.

affine transform

$$\bar{\mathbf{y}} = \mathbf{P}\mathbf{x} + \mathbf{r}, \tag{1}$$

where $\mathbf{P} \in \mathbb{R}^{M\times N}$ is the detection probability matrix with element $(i, j)$ equal to the probability of an event produced in voxel $j$ being detected by sinogram bin $i$, and $\mathbf{r} \in \mathbb{R}^{M\times 1}$ accounts for the presence of scatters and randoms in the data. The appropriate log-likelihood function is given by

$$L(\mathbf{y} \mid \mathbf{x}) = \sum_i (y_i \log \bar{y}_i - \bar{y}_i - \log y_i!), \tag{2}$$

where $\mathbf{y}$ is the measured data.

The ML reconstruction can be obtained by maximizing (2). However, the ML estimate can be very noisy because emission tomography is an ill-posed problem. Bayesian methods regularize the noise by using a prior probability distribution on the image. Most image priors use a Markov random field with a Gibbs distribution of the form

$$p(\mathbf{x}) = \frac{1}{Z} e^{-\beta U(\mathbf{x})}, \tag{3}$$

where $U(\mathbf{x})$ is the energy function, $\beta$ is the hyperparameter that controls the resolution of the reconstructed image, and $Z$ is a normalization constant. The Markovian properties of these distributions make them theoretically attractive as formalism for describing empirical local image properties, as well as computationally appealing. The energy function $U(\mathbf{x})$ often contains potentials defined on pair-wise cliques of neighboring voxels:

$$U(\mathbf{x}) = \sum_{j=1}^{N} \sum_{k\in\mathcal{N}_j} \kappa_{jk} V(x_j - x_k), \tag{4}$$

where $\mathcal{N}_j$ denotes the set of neighboring voxels of voxel $j$, $\kappa_{jk}$ are weighting factors, and $V(\cdot)$ is the potential function. A wide range of potential functions has been studied in the literature that attempt to produce local smoothing while not removing or blurring true boundaries or edges in the image. All have the basic property that they are monotonic nondecreasing functions of the absolute intensity difference $|x_j - x_k|$.

The potential function of a Gaussian prior is a quadratic function

$$V(x) = x^2. \tag{5}$$

It produces smooth images with very low probability of sharp transitions in intensity. In an attempt to increase the probability of sharp transitions, Bouman and Sauer [3] propose using the generalized $p$-Gaussian model where $V(x) = |x|^p$, $1 < p < 2$. An alternative function is the Huber prior in which $V(\cdot)$ is defined as [10]

$$V(x) = \begin{cases} \dfrac{x^2}{2\delta}, & |x| < \delta, \\ |x| - \dfrac{\delta}{2}, & |x| \geq \delta. \end{cases} \tag{6}$$

When $\delta$ is small, the Huber function approximates the absolute value function. It is probably the most edge-preserving prior with a convex potential function. Other potential functions with similar behavior are $V(x) = \log\cosh(\delta x)$ [6] and $V(x) = \delta^2[|x/\delta| - \log(1 + |x/\delta|)]$ [11]. In an attempt to produce even sharper intensity transitions, nonconvex functions have also been proposed. One example that we will study is the Geman-McClure prior [5], of which

$$V(x) = \frac{x^2}{x^2 + \delta^2}. \tag{7}$$

Figure 1 shows the potential functions of the Gaussian, Huber, and Geman-McClure priors. Note that in practice both the Huber prior and Geman-McClure prior can approach performance of the Gaussian prior by setting $\delta$ to be sufficiently large.

To demonstrate the difference between these potential functions, we show two images in Figure 2. For the Huber prior ($\delta \ll 1$) the two images are equally probable, while the Gaussian prior strongly favors the cone image (with an energy ratio of 50 : 1) and the Geman-McClure prior favors the disk image.

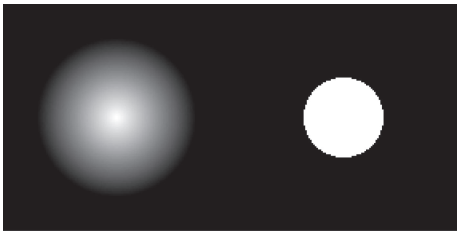

Figure 2: Images of a cone and a disk. The intensity in both images are between 0 and 1. See text for details.

Combine the likelihood function and prior distribution, the MAP reconstruction $\hat{\mathbf{x}}$ is found by maximizing the log-posterior density function:

$$\hat{\mathbf{x}} = \arg\max_{\mathbf{x}\geq 0} \left[L(\mathbf{y} \mid \mathbf{x}) - \beta U(\mathbf{x})\right]. \tag{8}$$

For priors with a convex potential function, (8) generally has a unique solution. When nonconvex potential functions are used, there may exist multiple local optima and the solution of most deterministic optimization algorithms will depend on the initial image.

### 3. METHODS

### 3.1. Computer simulation

We conduct computer simulations to study the effect of non-Gaussian priors on lesion detection. We simulate imaging of a prostate tumor using C-11 choline [12]. The simulated PET system has similar parameters as an ECAT HR+ clinical scanner (CPS, Knoxville, TN). It has 576 detectors forming a ring with radius 41.3 cm. The phantom has a body shape that is obtained from a patient image. The background has nearly uniform uptake of the radiotracer and has an attenuation coefficient of 0.0095 $\text{mm}^{-1}$. We place a round hot spot of different diameters (5 mm and 15 mm) at the center of the image to simulate a prostate tumor. Different pixel sizes are used in data generation and image reconstruction to introduce some model mismatch. For data generation, phantoms are represented by 256×256 2-mm square pixels, whereas 128×128 4.5-mm square pixels are used in reconstruction.

Figure 3 shows various phantom images that we used. Figure 3(a) is a fixed uniform background. Figures 3(b)–3(d) are three random backgrounds obtained by superimposing the background image in Figure 3(a) with a realization of lumpy backgrounds [13]. The lumpy backgrounds are modeled as

$$\text{LB} = \sum_{i=1}^{K} G(b, \sigma^2, \mathbf{r}_k), \tag{9}$$

where $G(b, \sigma^2, \mathbf{r}_k)$ is a Gaussian blob with variance $\sigma^2$ and height $b$ centering at a random location $\mathbf{r}_k$, and $K$ is a Poisson random variable. The mean of $K$ is set to 100. Two sets of $b$ and $\sigma$ are used: $b = 0.02$ and $\sigma = 32$ mm in Figure 3(b); $b = 0.1$ and $\sigma = 23$ mm in Figures 3(c) and 3(d). In Figure 3(d) we also add a hot region with activity to background ratio of 4 : 1 to mimic possible bladder uptake. In all four cases the mean activity of the background is about 0.20.

For each type of the background we generate three groups of data: background only, background with the 5-mm lesion, and background with the 15-mm lesion. Each group consists of 1000 independent identically distributed data sets. The expected total number of detected events is about 200 000. All data sets are independently reconstructed using a preconditioned conjugate gradient algorithm with the Gaussian prior, Huber prior, and Geman-McClure prior and different $\beta$ and $\delta$ values. Five hundred iterations are used to ensure effective convergence of the algorithm. All reconstructions start from a uniform image. For Geman-McClure prior, the reconstructed image may correspond to a local optimum of the log-posterior density function because the objective function is nonconvex.

### 3.2. Lesion detection

Detection of cancerous lesions is one major task of emission tomography. A standard methodology for studying lesion detectability is the receiver operating characteristic (ROC) study that compares true positive versus false positive rates for human observers for the task of lesion detection. Numerical observers based on signal-detection theory have been developed to mimic human performance [14]. For a given reconstructed image $\hat{\mathbf{x}}$, a linear numerical observer computes a test statistic (a scalar-valued decision variable), $\eta(\hat{\mathbf{x}})$, by

$$\eta(\hat{\mathbf{x}}) = \mathbf{t}'\hat{\mathbf{x}}, \tag{10}$$

where $\mathbf{t}$ is the observer template. The detection performance can be measured by the SNR of $\eta(\hat{\mathbf{x}})$, which is defined as

$$\begin{aligned}\text{SNR}^2\left[\eta(\hat{\mathbf{x}})\right] &= \frac{\left(E[\eta(\hat{\mathbf{x}}) \mid H_1] - E[\eta(\hat{\mathbf{x}}) \mid H_0]\right)^2}{\left(\text{var}\left[\eta(\hat{\mathbf{x}}) \mid H_1\right] + \text{var}\left[\eta(\hat{\mathbf{x}}) \mid H_0\right]\right)/2} \\ &= \frac{2(\mathbf{t}'\mathbf{z})^2}{\mathbf{t}'\Sigma_{\hat{\mathbf{x}}|H_1}\mathbf{t} + \mathbf{t}'\Sigma_{\hat{\mathbf{x}}|H_0}\mathbf{t}},\end{aligned} \tag{11}$$

where $H_0$ is the null hypothesis representing lesion absent, $H_1$ is the hypothesis representing lesion present, $\Sigma_{\hat{\mathbf{x}}|H_1}$ and $\Sigma_{\hat{\mathbf{x}}|H_0}$ are the conditional covariance matrices of $\hat{\mathbf{x}}$ under hypotheses of $H_1$ and $H_0$, respectively, and $\mathbf{z} \equiv E[\hat{\mathbf{x}} \mid H_1] - E[\hat{\mathbf{x}} \mid H_0]$ is the difference between the mean reconstructions under the two hypotheses. When $\eta(\hat{\mathbf{x}})$ is normally distributed, the area under the ROC curve (AUC) is related to the SNR by

$$\text{AUC} = \frac{1}{2}\left[1 + \text{erf}\left(\frac{\text{SNR}}{2}\right)\right], \tag{12}$$

where $\text{erf}(x)$ is the error function.

We use a channelized Hotelling observer (CHO), which has been shown to correlate with human performance [15–19]. The test statistic of CHO is

$$\eta(\hat{\mathbf{x}}) = \mathbf{z}'\mathbf{U}'\mathbf{K}^{-1}(\mathbf{U}\hat{\mathbf{x}} + \mathbf{n}), \tag{13}$$

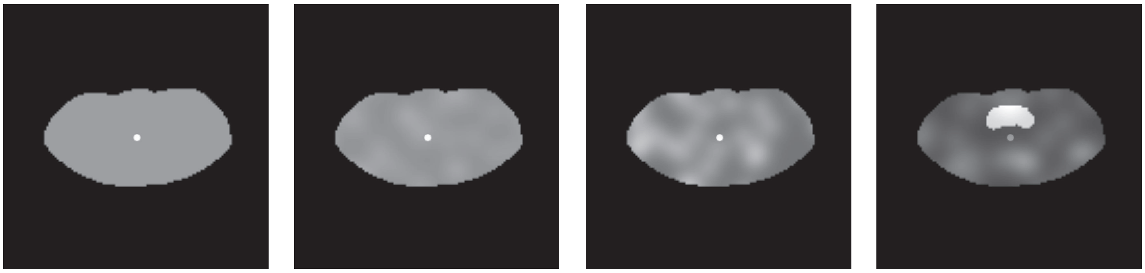

(a) (b) (c) (d)

FIGURE 3: The simulated phantom with the 15-mm lesion in four different backgrounds. (a) The uniform background; (b) a lumpy background of $b = 0.02$ and $\sigma = 32$ mm; (c) a lumpy background of $b = 0.1$ and $\sigma = 23$ mm; and (d) a lumpy background of $b = 0.1$ and $\sigma = 23$ mm with a hot region mimicking bladder uptake. The mean of the background in all cases is about 0.20.

where **U** denotes frequency-selective channels that mimic the human visual system, **n** is the internal channel noise that models the uncertainty in the human detection process [20] with zero mean and covariance $\mathbf{K}_N$, and **K** is the covariance of the channel outputs, that is,

$$\mathbf{K} = \frac{1}{2}\mathbf{U}(\Sigma_{\hat{\mathbf{x}}|H_1} + \Sigma_{\hat{\mathbf{x}}|H_0})\mathbf{U}' + \mathbf{K}_N. \tag{14}$$

In this work the channel functions are the differences of four Gaussian functions with standard deviations $\sigma = 2.653, 1.592, 0.995$, and 0.573, respectively [21]. The internal noise is modeled as uncorrelated noise with $\mathbf{K}_N = \text{diag}[0.15\sigma_i^2 + 0.25\max_i\{\sigma_i^2\}]$, where $\sigma_i^2$ is the data variance in the $i$th channel output [22]. Monte Carlo reconstructions are used to calculate the expectation of the reconstruction and covariance matrices. The SNR of CHO is then calculated by

$$\text{SNR}^2\left[\eta(\hat{\mathbf{x}})\right] = \mathbf{z}'\mathbf{U}'\mathbf{K}^{-1}\mathbf{U}\mathbf{z} \tag{15}$$

because the SNR calculated from (15) is meaningful only when $\eta(\hat{\mathbf{x}})$ is normally distributed, we also calculate AUCs from empirical ROC curves using numerical integration.

### 3.3. Quantification performance

Another clinical task in emission tomography is to quantify the uptake of radioactive tracer in a region of interest (ROI). This can be written as

$$\eta_Q(\hat{\mathbf{x}}) = \mathbf{t}'\hat{\mathbf{x}}, \tag{16}$$

where **t** is the indicator function of the ROI, that is, $t_j = 1$ if voxel $j$ is inside the ROI, and $t_j = 0$ otherwise. The accuracy of the quantification can be measured by the bias and variance of $\eta_Q(\hat{\mathbf{x}})$ as

$$\begin{aligned}\text{bias}(\eta_Q) &= \mathbf{t}'\mathbf{x} - \mathbf{t}'E[\hat{\mathbf{x}}],\\ \text{var}(\eta_Q) &= \mathbf{t}'\Sigma_{\hat{\mathbf{x}}|H_1}\mathbf{t},\end{aligned} \tag{17}$$

where **x** denotes the true tracer uptake. Note that the ROI quantification is only performed on images in which a lesion is known to be present.

We use the bias versus variance tradeoff curve to evaluate the quantification performance. The above equations are calculated from Monte Carlo reconstructions.

### 4. RESULTS

Figures 4 and 5 show examples of reconstructed images with different priors. For all priors the reconstructed images become less noisy as we increase the hyperparameter $\beta$. The images reconstructed with the Gaussian prior have blurred edges, whereas the images reconstructed with the Huber prior and Geman-McClure prior tend to form piece-wise constant regions.

Figure 6 shows the variation of AUC as a function of $\beta$ and $\delta$ for detecting the 15-mm lesion in the lumpy background shown in Figure 3(c). In this case, the optimum parameters for the Gaussian, Huber, and Geman-McClure priors are $\beta = 30$, ($\beta = 10$, $\delta = 0.1$), and ($\beta = 0.3$, $\delta = 0.1$), respectively. Comparing to the results of the fixed background (not shown), we found that lumpy backgrounds reduce lesion detectability at low resolution (large $\beta$) and slightly decrease the optimum $\beta$ value for lesion detection.

To give a fair comparison between different priors, we choose to compare the maximum SNR and maximum AUC of each prior. The results are shown in Figure 7. The error bars are computed using a bootstrap method. In all detection studies, the contrasts of the 5-mm and 15-mm lesions are 3 and 0.9, respectively, which are selected to obtain a reasonable detectability (AUC $\approx$ 0.9). In each case, SNR and AUC give similar ranking for the optimum performances of the three priors. No statistically significant advantage is found for non-Gaussian priors. For lesions with lower contrast, we expect the performances of the three priors to be even closer. We notice that some differences in AUC do not correspond well to the differences in SNR (see Figures 7(c) and 7(d)), which indicates that the test statistics of the numerical observer does not follow a Gaussian distribution.

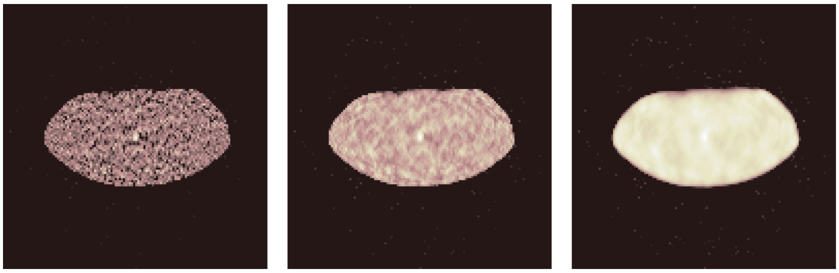

(a) Gaussian prior

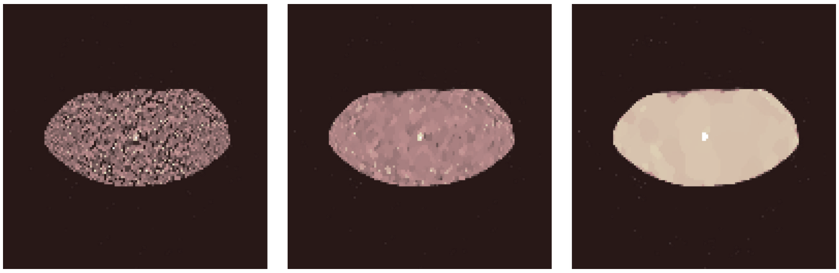

(b) Huber prior

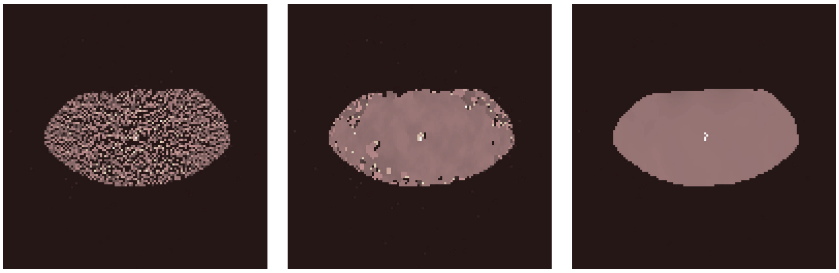

(c) Geman-McClure prior

Figure 4: Reconstructed images of one noisy data set with a fixed background. The three images in each group are reconstructed with different $\beta$.

Figure 8 shows the bias versus variance tradeoff curves for quantification. The contrast of the lesion is 3.0 for the 5-mm lesion and 0.9 for the 15-mm lesion, respectively. The ROIs were obtained from the original phantom image. Bias and standard deviation are normalized to the total activity inside each ROI. Here we plot all the cases. It is interesting to see that Gaussian prior seems to set a lower bound for the Huber and Geman-McClure priors (except in Figure 8(h)). Considering that both Huber and Geman-McClure priors include Gaussian prior as a special case, no improvement in ROI quantification is found by using the Huber or Geman-McClure prior. In many cases, the performance of the non-Gaussian priors is much worse than that of the Gaussian prior, indicating that hyperparameter selection is more important for non-Gaussian priors. In Figures 8(b), 8(d), 8(f) there is a kink in the Gaussian bias-variance curve at low noise levels. This is because at such low resolution the reconstructed background is no longer uniform, but forms a dome, which artificially increases the activity inside the ROI and, hence, reduces the bias. The decrease in bias at low noise levels in Figures 8(g) and 8(h) is mostly caused by the spill-over effect of the nearby hot region ("bladder").

We also studied lesions with higher contrast (9 for the 5-mm lesion and 2.7 for the 15-mm lesion, respectively) and found very similar results [9]. To investigate the bias-variance tradeoff for large regions, we quantify the total activity in the hot "bladder" in Figure 3(d). The results are shown in Figure 9. Even for this large region with 4 : 1 activity ratio, we do not see any advantage of using the Huber and Geman-McClure priors.

### 5. CONCLUSION AND DISCUSSION

We have compared the performance of three representative priors for lesion detection and ROI quantification. The Gaussian prior is the most commonly used prior in emission reconstruction; the Huber prior is probably the most

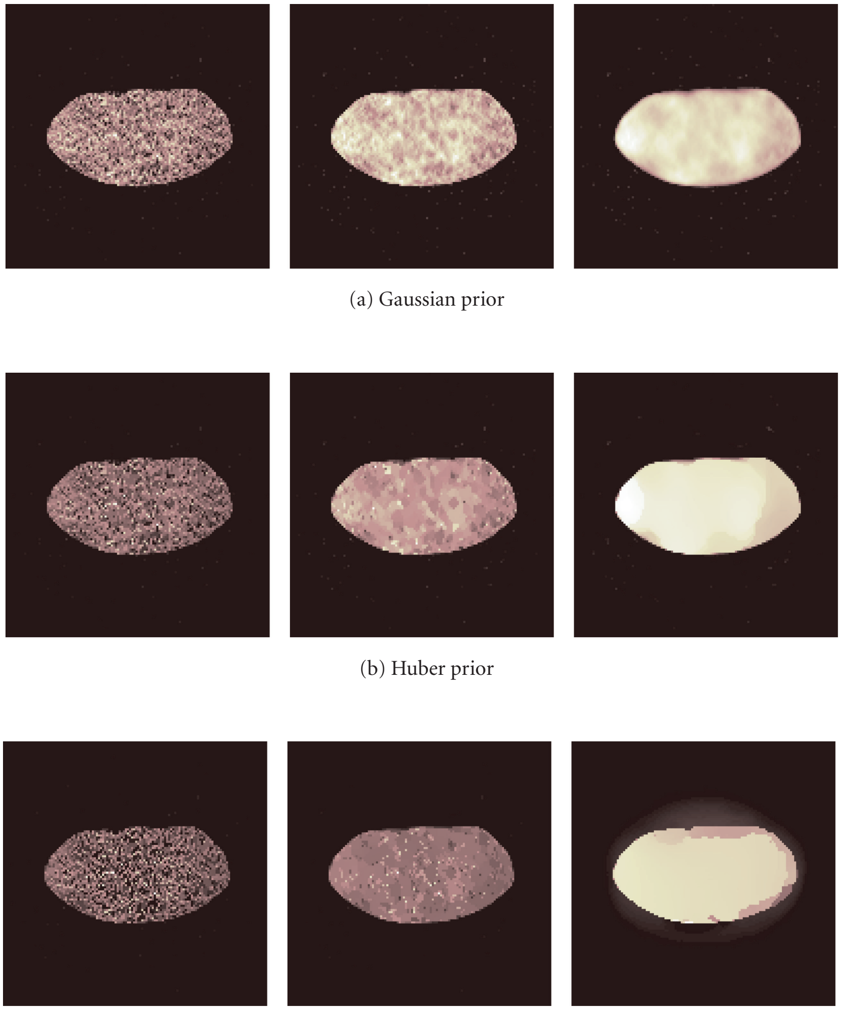

(a) Gaussian prior

(b) Huber prior

(c) Geman-McClure prior

Figure 5: Reconstructed images of one noisy data set with the lumpy background shown in Figure 3(c). The three images in each group are reconstructed with different $\beta$.

edge-preserving prior among all priors with a convex potential function; and the Geman-McClure is a typical edge-preserving prior with a nonconvex potential function. Note that both the Huber and Geman-McClure priors can approach the Gaussian prior by setting $\delta$ to be sufficiently large. Thus we focus on whether the Huber and Geman-McClure priors can outperform the Gaussian prior. In all the cases that we have tested, we have not observed any significant improvement. The results show that for the detection and quantification tasks that are considered here, the Gaussian prior is as effective as the more complex non-Gaussian priors.

We should note that while we have investigated each prior with a range of $\beta$ and $\delta$ values, it is still possible that the results may not reflect the best performance for non-Gaussian priors because of the lack of theoretical guidance on the hyperparameter selection for non-Gaussian priors. Nonetheless, the results indicate that hyperparameter selection is extremely important for non-Gaussian priors. For the Geman-McClure prior, the simulation results presented here may not correspond to the global maximum of the log posterior distribution because the objective function is nonconcave. It is possible that the performance of Geman-McClure prior may be improved by using deterministic or stochastic annealing techniques at an expense of increased computational cost [23].

In this paper we used a channelized Hotelling observer for lesion detection and a simple ROI estimator for quantification because of their popularity. It is possible that some results might change if different observers or estimators were used. In ROI quantification we defined ROI using the original phantom data. If an ROI is to be delineated on the reconstructed image, the error in ROI delineation will also affect the ROI quantification [24]. Such effect is not included in this study. We expect that with the recent development of combined PET/CT and SPECT/CT scanners, high-resolution

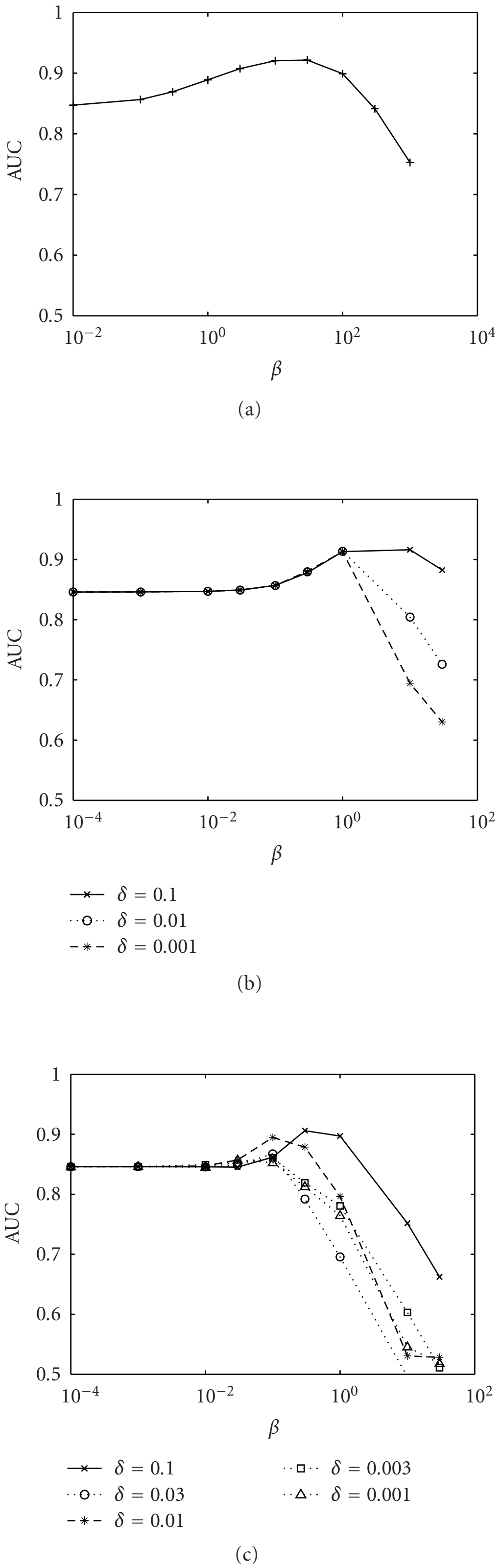

Figure 6: Plots of AUCs for detecting the 15-mm lesion in the lumpy background shown in Figure 3(c) as a function of the prior parameters: (a) Gaussian prior; (b) Huber prior; and (c) Geman-McClure prior.

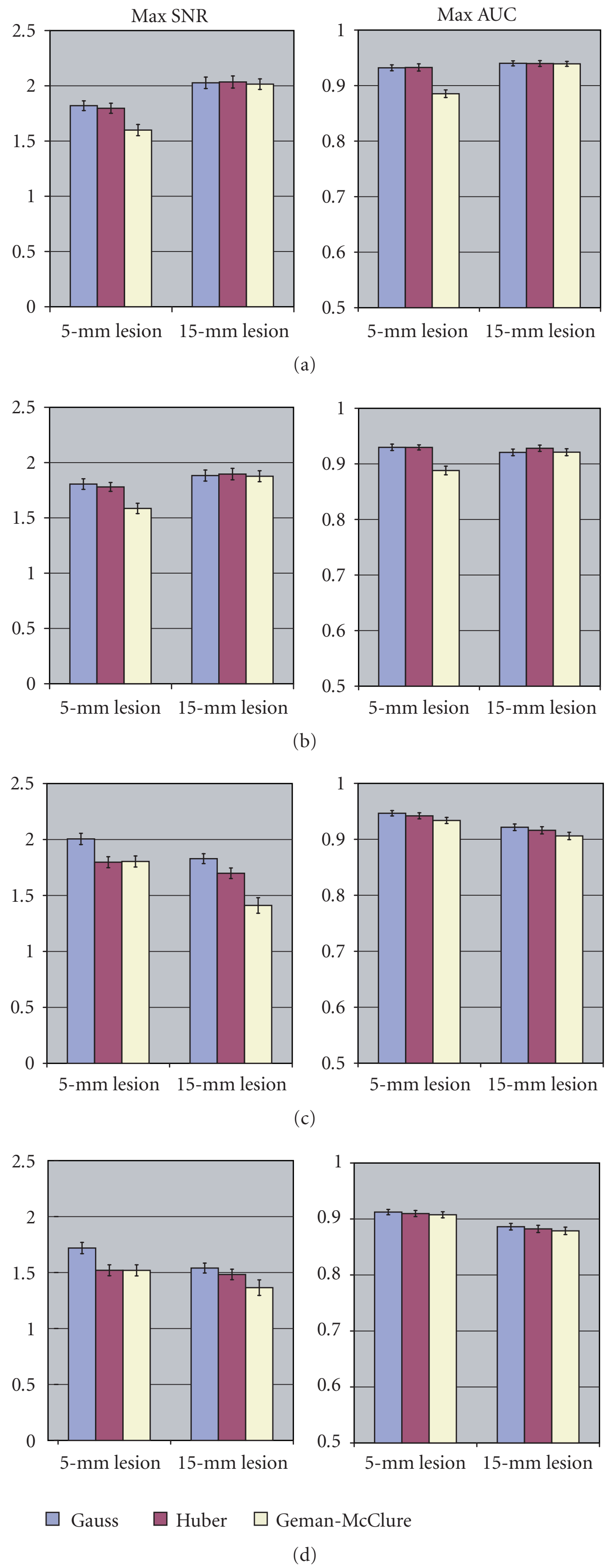

Figure 7: The maximum SNR and maximum AUC for detecting a lesion in different backgrounds. (a) Fixed background; (b) lumpy backgrounds in Figure 3(b); (c) lumpy backgrounds in Figure 3(c); and (d) lumpy backgrounds in Figure 3(d).

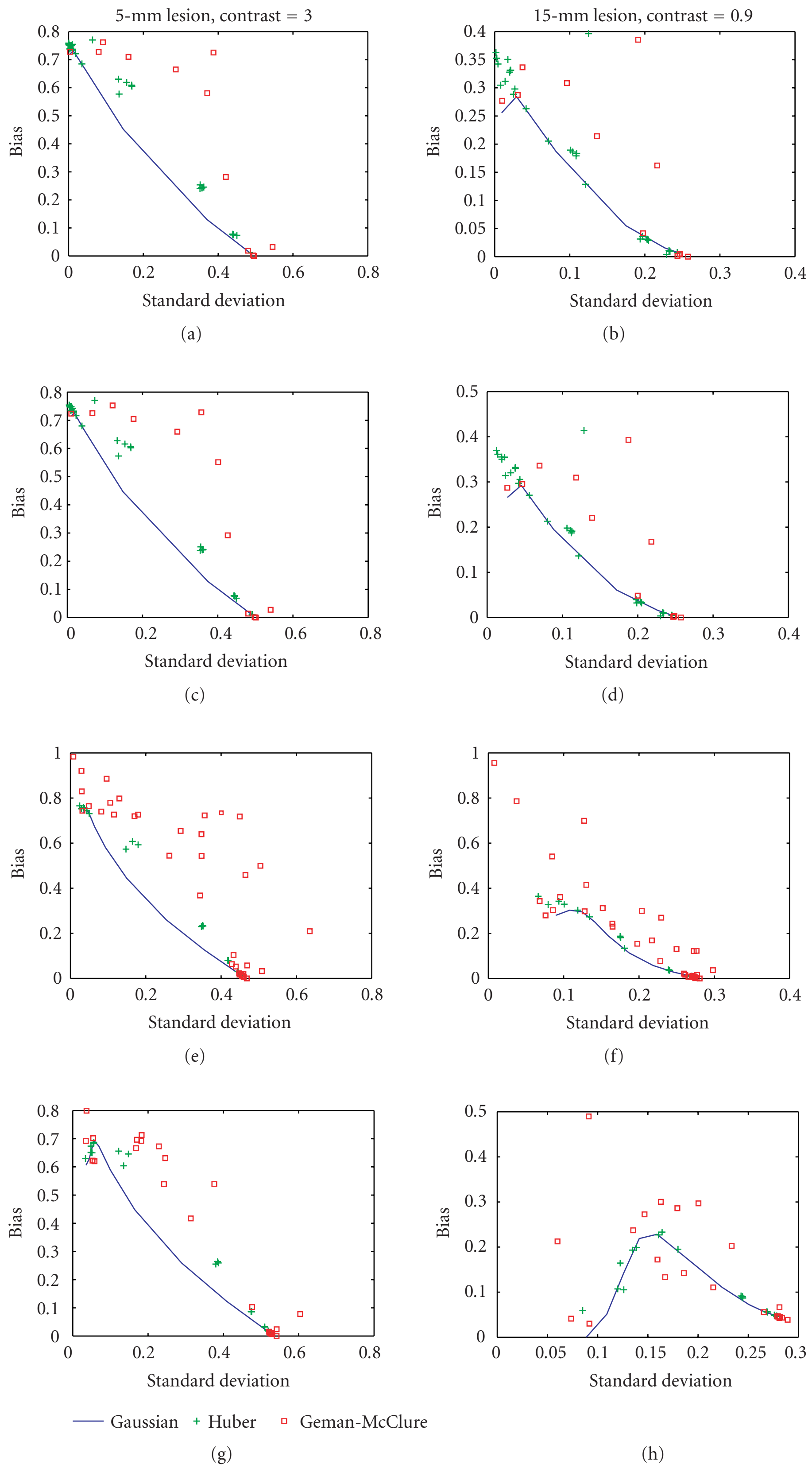

FIGURE 8: The bias versus standard deviation tradeoff for quantification of total uptake of a tumor in different backgrounds. (a) and (b) Fixed uniform background; (c) and (d) lumpy backgrounds in Figure 3(b); (e) and (f) lumpy backgrounds in Figure 3(c); and (g) and (h) lumpy backgrounds in Figure 3(d).

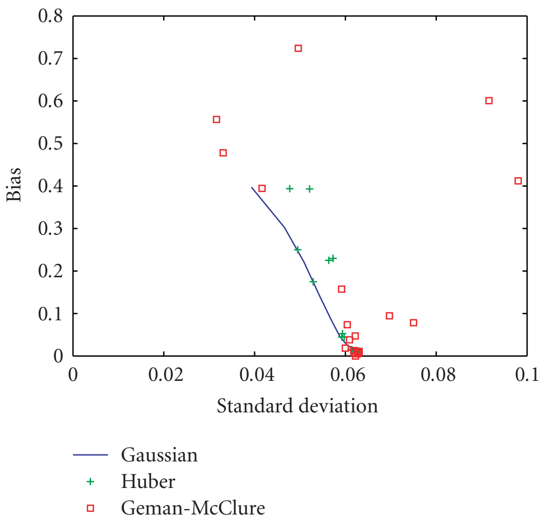

Figure 9: The bias versus standard deviation tradeoff for quantification of the large hot region ("bladder") in Figure 3(d).

anatomical images will help to reduce the error in ROI definition.

### ACKNOWLEDGMENT

This work is supported by National Institutes of Health under Grant R01 EB000194.

### REFERENCES

[1] L. Shepp and Y. Vardi, "Maximum likelihood reconstruction for emission tomography," *IEEE Transactions on Medical Imaging*, vol. 1, no. 2, pp. 113–122, 1982.

[2] J. A. Fessler and A. O. Hero III, "Penalized maximum-likelihood image reconstruction using space-alternating generalized EM algorithms," *IEEE Transactions on Image Processing*, vol. 4, no. 10, pp. 1417–1429, 1995.

[3] C. A. Bouman and K. Sauer, "A unified approach to statistical tomography using coordinate descent optimization," *IEEE Transactions on Image Processing*, vol. 5, no. 3, pp. 480–492, 1996.

[4] K. Lange and R. Carson, "EM reconstruction algorithms for emission and transmission tomography," *Journal of Computer Assisted Tomography*, vol. 8, no. 2, pp. 306–316, 1984.

[5] S. Geman and D. McClure, "Bayesian image analysis: an application to single photon emission tomography," in *Proceedings of the Statistical Computing Section of the American Statistical Association*, pp. 12–18, Washington, DC, USA, 1985.

[6] P. J. Green, "Bayesian reconstructions from emission tomography data using a modified EM algorithm," *IEEE Transactions on Medical Imaging*, vol. 9, no. 1, pp. 84–93, 1990.

[7] H. C. Gifford, T. H. Farncombe, and M. A. King, "Ga-67 tumor detection using penalized-EM with nonanatomical regularizers," in *Proceedings of the IEEE Nuclear Science Symposium and Medical Imaging Conference (NSS-MIC '02)*, vol. 3, pp. 1397–1401, Norfolk, Va, USA, November 2002.

[8] J. Nuyts and C. Michel, "Performance of the relative difference prior for hot lesion detection in whole-body PET/CT: an evaluation with numerical and real observers," in *Proceedings of the IEEE Nuclear Science Symposium and Medical Imaging Conference (NSS-MIC '05)*, pp. 2155–2159, Wyndham El Conquistador, Puerto Rico, USA, October 2005.

[9] J. Qi, "Investigation of lesion detection and quantitation in MAP reconstruction with non-Gaussian priors," in *Proceedings of the IEEE Nuclear Science Symposium and Medical Imaging Conference (NSS-MIC '05)*, pp. 1704–1708, Wyndham El Conquistador, Puerto Rico, USA, October 2005.

[10] P. J. Huber, *Robust Statistics*, John Wiley & Sons, New York, NY, USA, 1981.

[11] K. Lange, "Convergence of EM image reconstruction algorithms with Gibbs smoothing," *IEEE Transactions on Medical Imaging*, vol. 9, no. 4, pp. 439–446, 1990, correction: vol. 10, no. 2, p. 228, 1991.

[12] T. Hara, N. Kosaka, and H. Kishi, "PET imaging of prostate cancer using carbon-11-choline," *Journal of Nuclear Medicine*, vol. 39, no. 6, pp. 990–995, 1998.

[13] J. P. Rolland and H. H. Barrett, "Effect of random background inhomogeneity on observer detection performance," *Journal of the Optical Society of America A*, vol. 9, no. 5, pp. 649–658, 1992.

[14] H. H. Barrett, J. Yao, J. Rolland, and K. Myers, "Model observers for assessment of image quality," *Proceedings of the National Academy of Sciences of the United States of America*, vol. 90, no. 21, pp. 9758–9765, 1993.

[15] C. K. Abbey, H. H. Barrett, and D. W. Wilson, "Observer signal-to-noise ratios for the ML-EM algorithm," in *Medical Imaging 1996: Image Perception*, vol. 2712 of *Proceedings of SPIE*, pp. 47–58, Newport Beach, Calif, USA, February 1996.

[16] D. de Vries, M. A. King, E. Soares, B. Tsui, and C. Metz, "Effects of scatter subtraction on detection and quantitation in hepatic SPECT," *Journal of Nuclear Medicine*, vol. 40, no. 6, pp. 1011–1023, 1999.

[17] T. Narayan and G. Herman, "Prediction of human observer performance by numerical observers: an experimental study," *Journal of the Optical Society of America A*, vol. 16, no. 3, pp. 679–693, 1999.

[18] S. D. Wollenweber, B. M. W. Tsui, D. S. Lalush, E. C. Frey, K. J. LaCroix, and G. T. Gullberg, "Comparison of Hotelling observer models and human observers in defect detection from myocardial SPECT imaging," *IEEE Transactions on Nuclear Science*, vol. 46, no. 6, pp. 2098–2103, 1999.

[19] H. Gifford, M. A. King, D. de Vries, and E. Soares, "Channelized Hotelling and human observer correlation for lesion detection in hepatic SPECT imaging," *Journal of Nuclear Medicine*, vol. 41, no. 3, pp. 514–521, 2000.

[20] J. Yao and H. H. Barrett, "Predicting human performance by a channelized Hotelling observer model," in *Mathematical Methods in Medical Imaging*, vol. 1768 of *Proceedings of SPIE*, pp. 161–168, San Diego, Calif, USA, July 1992.

[21] M. A. King, D. de Vries, and E. Soares, "Comparison of the channelized Hotelling and human observers for lesion detection in hepatic SPECT imaging," in *Medical Imaging 1997: Image Perception*, vol. 3036 of *Proceedings of SPIE*, pp. 14–20, Newport Beach, Calif, USA, February 1997.

[22] J. Oldan, S. Kulkarni, Y. Xing, P. Khurd, and G. Gindi, "Channelized hotelling and human observer study of optimal smoothing in SPECT MAP reconstruction," *IEEE Transactions on Nuclear Science*, vol. 51, no. 3, pp. 733–741, 2004.

[23] S. Geman and D. Geman, "Stochastic relaxation, Gibbs distributions, and the Bayesian restoration of images," *IEEE Transactions on Pattern Analysis and Machine Intelligence*, vol. 6, no. 6, pp. 721–741, 1984.

[24] R. G. Wells, H. C. Gifford, and M. A. King, "Impact of ROI definition on estimator performance on FBP reconstructed Ga-67 SPECT images," *IEEE Transactions on Nuclear Science*, vol. 47, no. 3, pp. 1210–1217, 2000.

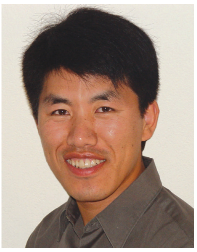

**Jinyi Qi** received the B.Eng. degree (with highest honor) from Tsinghua University in Beijing in 1993, and the M.S. and Ph.D. degrees from the University of Southern California in 1997 and 1998, respectively, all in electrical engineering. He was recognized with the 2001 Young Investigator Medical Image Science Award by the IEEE Nuclear and Plasma Science Society for contributions to the analysis of Bayesian image reconstruction algorithms, and for the development of high-resolution 3D Bayesian image reconstruction methods for animal PET scans. Currently he is an Assistant Professor in the Department of Biomedical Engineering at the University of California, Davis, and is also a Faculty Scientist in the Department of Functional Imaging at Lawrence Berkeley National Laboratory. His research interests include statistical image reconstruction, medical image processing, image quality evaluation, and imaging system optimization.

